# NLR (neutrophil to lymphocyte ratio), PLR (platelet to lymphocyte ratio), and SII (systemic immune-inflammation index) reflect disease activity and renal remission in patients with lupus nephritis

**DOI:** 10.3389/fimmu.2025.1646276

**Published:** 2025-09-30

**Authors:** Xiaohui Zhang, Yuan Chen, Yong Fan, Dai Gao, Zhuoli Zhang

**Affiliations:** ^1^ Department of Rheumatology and Clinical Immunology, Peking University First Hospital, Beijing, China; ^2^ National Clinical Research Center for Skin and Immune Diseases, Beijing, China

**Keywords:** neutrophil to lymphocyte ratio, platelet to lymphocyte ratio, systemic immune-inflammation index, lupus nephritis, disease activity, renal remission

## Abstract

**Objective:**

To evaluate the value of NLR (neutrophil to lymphocyte ratio), PLR (platelet to lymphocyte ratio), and SII (systemic immune-inflammation index) in reflecting disease activity and induction therapy remission in patients with lupus nephritis (LN).

**Methods:**

Active LN patients from STAR cohort were enrolled. We analyzed the trends of complete blood count parameters with Generalized Estimated Equation. Bivariate correlation analyses, Chi-square tests, *t*-tests and logistic regression were employed to assess variable associations and identify prognostic factors for LN remission.

**Results:**

310 active LN patients were enrolled in the study. All patients had active lupus with SLEDAI-2K 17.1 ± 6.1, median 24h-Urine Protein (UTP) level of 3.1 (1.5, 5.4) g. During the 12-month follow-up of induction therapy, NLR and PLR showed a decreasing trend. Both baseline NLR and SII were positively correlated with baseline UTP and serum creatinine (SCr) levels (*r* = 0.112-0.148, p< 0.05 for all). Patients with hematuria [4.8 (3.1, 8.1) *vs*. 4.0 (2.6, 6.5), p = 0.024] and pyuria [5.4 (3.4, 8.8) *vs*. 3.8 (2.6, 6.6), p < 0.001] had significantly higher baseline NLR. 159 (51.3%) patients performed kidney biopsy, and baseline NLR and SII were positively correlated with the activity index (AI) score of renal pathology (NLR: *r* = 0.244, p=0.013; SII: *r* = 0.199, p=0.043). Furthermore, the changes of NLR and SII were also positively correlated with changes in UTP and SCr during 6 and 12 months (*r* = 0.143-0.175, p<0.05 for all). Nevertheless, neither of baseline NLR, PLR, or SII could predict renal remission at 6 months.

**Conclusion:**

Our findings suggested that NLR and SII were valuable indicators of disease activity in LN, correlating with UTP, SCr and AI score of renal pathology. NLR, PLR and SII provided us a quick, simple and cost-effective supervision way in monitoring and managing LN patients.

## Introduction

Systemic lupus erythematosus (SLE) is a complex autoimmune disease characterized by involvement of multiple organ systems. Lupus nephritis (LN), affecting about 40% of SLE patients, is associated with significant morbidity and mortality. If left untreated or inadequately managed, LN can lead to severe complications such as chronic kidney disease, end-stage renal disease, or even death (1). While routine urine analysis and serum creatinine levels can provide some insight into renal injury, kidney biopsy remains the definitive method for assessing disease activity in LN. However, not all patients undergo this invasive procedure. Although markers such as complement levels and anti-double-stranded DNA (anti-dsDNA) antibodies are traditionally employed as clinical indicators of lupus activity, they are expensive and not readily accessible in urgent clinical settings. This underscores the critical need for the development of rapid and convenient biomarkers that can effectively gauge disease activity in SLE, particularly for LN patients.

Recent studies have indicated that NLR (neutrophil to lymphocyte ratio), PLR (platelet to lymphocyte ratio), and SII (systemic immune-inflammation index) can serve as valuable tools for assessing inflammation and systemic immune responses (2–5). They can help differentiate between active and inactive disease states, as well as to distinguish infection from disease exacerbation in many rheumatic diseases (5–7). And some studies also found that NLR and PLR contribute to SLE disease activity monitoring and infection differential diagnosis (8–12). Despite the promising potential of these markers, there remains a paucity of research specifically addressing their implications in LN patients. This study aims to explore the significance of NLR, PLR, and SII in relation to disease activity and prognosis in the context of LN.

## Materials and methods

### Patients

All patients in the study were from the Treat SLE to Target (STAR) cohort, a prospective longitudinal cohort that has been running in Peking University First Hospital since 2007. All patients met 1999 American College of Rheumatology (ACR) criteria, the 2012 Systemic Lupus International Collaborating Clinics criteria, or the 2019 EULAR/ACR classification criteria for SLE. Eligibility for participation in this study was strictly defined, requiring candidates to fulfill the 2003 International Society of Nephrology and Renal Pathology Society (ISN/RPS) classification criteria for lupus nephritis (LN) while exhibiting active LN that necessitated induction therapy. The exclusion criteria were as follows: (1) age < 18 years; (2) pregnancy; (3) urinary protein < 0.5g/24 hours before initiating remission induction therapy; (4) combined with acute infections, tumors, thrombotic microangiopathy, other critical organ diseases such as heart disease, liver and kidney diseases, as well as blood system diseases; (5) those who have received blood transfusion therapy (excluding plasma) within the past 3 months before undergoing blood routine examination; (6) cannot provide data regarding NLR, PLR and SII. This research adheres to the ethical standards outlined in the Helsinki Declaration, having received prior approval from the ethics committee of Peking University First Hospital (2017-1284). Informed consent was obtained from each patient at enrolment.

### Data collection

The demographics, clinical data, and laboratory data were recorded. The time point of initiating induction therapy for LN was defined as the baseline of this study. Baseline urine and blood samples were drawn before the LN induction treatment. Blood routine was tested by an automatic blood cell analyzer (Beckman coulter DxH 800). NLR was calculated as the ratio of neutrophil count to lymphocyte count, PLR as the ratio of platelet to lymphocyte count. SII was calculated as the product of the platelet count and the neutrophil count divided by the lymphocyte count. The disease activity of SLE was assessed at each visit using SLE disease activity index 2000 (SLEDAI-2K). Renal SLEDAI (rSLEDAI) was the sum of four urine components including proteinuria (>0.5 g per 24 h), hematuria (>5 red blood cells per high-power field), pyuria (>5 white blood cells per high-power field), or casts (heme, granular, or red blood cells). The definition of LN types was based on International Society of Nephrology/Renal Pathology Society (ISN/RPS) 2003 lupus nephritis pathological standard. The National Institutes of Health (NIH) activity index (AI) and chronicity index (CI) were obtained for renal pathology. The ranges were 0–24 for AI and 0–12 for CI, respectively.

The renal outcomes were evaluated at 6 and 12 months. Complete renal remission (CRR) required (1) proteinuria <0.5 g/24 hours; (2) serum creatinine (SCr) within 15% changes from baseline. Total renal remission (TRR) at 6 and 12 months, defined as ≥50% reduction in proteinuria to subnephrotic levels, and SCr within 15% changes.

### Statistical analysis

Categorical variables were presented as counts and percentages. Normally distributed data are expressed as mean ± standard deviation, while non-normally distributed variables are expressed as median and interquartile range. We analyzed the trends of complete blood count parameters with Generalized Estimated Equation. Bivariate correlation analyses were conducted to evaluate the associations between variables. The strengths of the correlations between NLR, PLR, and SII and various disease parameters—including clinical measures (e.g., proteinuria, serum creatinine), disease activity scores (SLEDAI-2K), and histopathological indices (activity and chronicity index)—were assessed using Spearman’s or Pearson’s correlation coefficients. Categorical variables were compared using Chi-square tests or Fisher’s exact tests as appropriate. For continuous variables, comparisons were carried out with Student’s *t*-test or Mann-Whitney U test, depending on the distribution. Logistic regression analysis was performed to identify prognostic factors associated with lupus nephritis remission. All statistical analyses were conducted using IBM SPSS Statistics for Windows, version 26 (IBM Corp., Armonk, NY, USA). Graphs were generated using GraphPad Prism version 8.0.2. All tests were two-sided, with p<0.05 considered statistically significant.

## Results

### Baseline clinical characteristics of enrolled LN patients

A total of 310 active LN patients were included in this study. Their clinical characteristics are presented in **Supplementary Table 1**. The median age was 35.9 years, with 264 (85.2%) being women. The median duration of SLE was 2.1 years. All patients were positive for antinuclear antibodies (ANA), and 83.2% were positive for anti-double-stranded DNA (anti-dsDNA). Autoantibodies targeting SSA were found in 58.1% of patients, nRNP in 42.3%, Sm in 33.9%, rRNP in 28.7%, SSB in 16.8%, and anti-neutrophil cytoplasmic antibodies (ANCA) in 9.4%. Additionally, 48 patients (15.5%) tested positive for lupus anticoagulants, 32 (10.3%) had anticardiolipin antibodies, and 45 (14.5%) had antibodies against 2 Glycoprotein I.

All patients had active lupus with SLEDAI-2K 17.1 ± 6.1, median UTP level of 3.1 (1.5, 5.4) g, serum Alb 28.0 ± 6.5 g/L and SCr 74.0 (61.2, 100) mol/L. 163 (52.6%) patients were treatment-naive before enrolment. 159 (51.3%) patients performed kidney biopsy, and IV type was most common.

### Changes in complete blood counts and inflammatory markers (NLR, PLR, SII)

**Figure 1:**
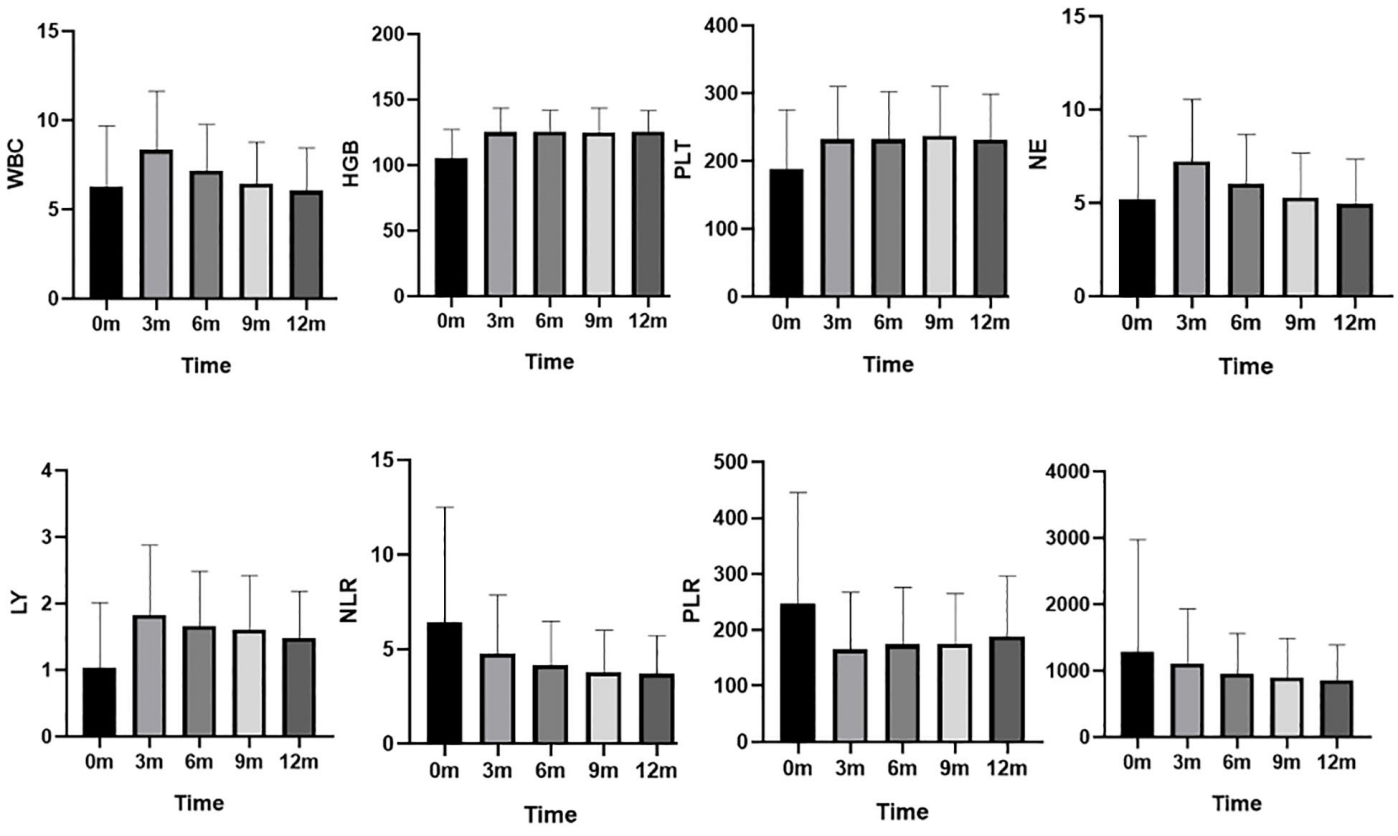
Changes in complete blood counts and NLR, PLR, SII during the 12-month follow-up. WBC (×10_9_/L): white blood cell counts; HGB (g/L): hemoglobin level ; PLT (×10_9_/L): platelets counts; NE (×10_9_/L):neutrophil counts; LY (×10_9_/L):Lymphocyte counts; NLR: neutrophil to lymphocyte ratio , PLR: platelet to lymphocyte ratio, and SII: systemic immune-inflammation index.

Changes in complete blood counts and NLR, PLR, SII during the 12-month follow-up are presented in **Table 1** and **Figure 1**. Lymphocyte counts increased from 0.9 (0.6, 1.3) at baseline to 1.5 (1.1, 2.3) at 3 months and remained stable at 6, 9, and 12 months. White blood cell (WBC) counts and neutrophil counts showed a trend of initially increasing and then decreasing back to baseline levels. The NLR decreased from 4.5 (3.0, 7.6) at baseline to 3.9 (2.8, 5.7) at 3 months, 3.6 (2.7, 4.8) at 6 months, 3.3 (2.4, 4.3) at 9 months, and 3.1 (2.4, 4.5) at 12 months, indicating a sustained improvement in the inflammatory state. The PLR also showed a significant decrease compared to baseline. In contrast, the trend in the SII was not as pronounced. Additionally, hemoglobin and platelet counts improved at 3 months and remained stable around 125 g/L and 230 x 10^9^/L, respectively, indicating sustained improvement.

**Table 1:** Changes in complete blood counts and inflammatory markers (NLR, PLR, SII) during 12-month follow-up of SLE patient.

Times	WBC	HGB	PLT	NE	LY	NLR	PLR	SII
0 month	6.3±3.4	105.1±22.2	188.6±86.7	4.5 (2.6, 6.9)	0.9 (0.6, 1.3)	4.5 (3.0, 7.6)	191.3 (130.7, 307.3)	796.1 (427.1, 1462.1)
3 months	8.3±3.3^*^	125.2±18.4^*^	232.5±77.5^*^	6.8 (5.1, 9.0)^*^	1.5 (1.1, 2.3)^*^	3.9 (2.8, 5.7)^*^	136.5 (95.5, 212.1)^*^	882.0 (628.1, 1312.9)
6 months	7.1±2.6^*^	125.9±16.2^*^	232.4±70.1^*^	5.8 (4.2, 7.6)^*^	1.5 (1.1, 2.2)^*^	3.6 (2.7, 4.8)^*^	149.3 (104.1, 209.2)^*^	815.4 (571.2, 1178.6)
9months	6.4±2.3	124.9±18.8^*^	237.3±73.1^*^	4.9 (3.7, 6.5)	1.4 (1.1, 2.0)^*^	3.3 (2.4, 4.3)^*^	163.6 (110.0, 223.5)^*^	754.2 (542.9, 1030.0)^*^
12 months	6.1±2.4	125.7±15.8^*^	232.0±66.8^*^	4.6 (3.3, 6.1)	1.3 (1.0, 1.9)^*^	3.1 (2.4, 4.5)^*^	163.6 (123.6, 230.0)^*^	716.6 (542.3, 1061.1)^*^
P_trend_ values	0.001	0.001	0.001	0.001	0.001	0.001	0.001	0.003

* p<0.05 compared 0 month.

### Correlation between NLR, PLR, SII and LN disease activity

We performed correlation analyses between baseline levels of NLR, PLR, and SII and key laboratory parameters of lupus nephritis (**Table 2**). NLR and SII showed weak but statistically significant positive correlations with UTP (NLR: *r* = 0.131, p=0.021; SII: *r* = 0.148, p=0.009) and SCr levels (NLR: *r* = 0.139, p=0.015; SII: *r* = 0.112, p=0.049). Furthermore, PLR shows a significant negative correlation with serum albumin (Alb) levels *(r* = -0.151, p=0.008). Patients with hematuria [4.8 (3.1, 8.1) *vs*. 4.0 (2.6, 6.5), p = 0.024] and pyuria [5.4 (3.4, 8.8) *vs*. 3.8 (2.6, 6.6), p < 0.001] had significantly higher NLR. However, no significant correlations were found between NLR, PLR, SII and complement levels, SLEDAI, or rSLEDAI.

**Table 2:** Correlation between baseline NLR, PLR, SII and LN indicators.

Parameters of LN	NLR		PLR		SII	
	*r*	*P*	*r*	*P*	*r*	*P*
UTP (g)	0.131	0.021	0.081	0.156	0.148	0.009
SCr (μmol/L)	0.139	0.015	0.060	0.294	0.112	0.049
Alb (g/L)	-0.080	0.163	-0.151	0.008	-0.070	0.219
C3 (g/L)	-0.051	0.374	-0.085	0.310	-0.027	0.630
C4 (g/L)	-0.009	0.872	0.030	0.593	-0.018	0.751
SLEDAI	0.048	0.399	0.117	0.040	0.018	0.753
rSLEDAI	0.081	0.154	0.025	0.663	0.049	0.385

We analyzed the relationship between the changes (Δ) in NLR, PLR and SII with various clinical indicators of LN before and after treatment. The results indicate that significant positive correlations existed between ΔNLR and ΔSII with ΔUTP and ΔSCr at both 6 and 12 months (r=0.143-0.175, p all<0.05) (**Table 3**), suggesting a potential relationship between systemic inflammatory markers and renal function indicators in patients with LN. Conversely, changes in Alb levels showed significant negative correlations with ΔSII (r=-0.211, p=0.001). Changes in the rSLEDAI index did not show significant correlations with any of the inflammatory markers (**Table 3**).

**Table 3:** Correlation between changes of NLR, PLR, SII and changes of LN indicator.

Times		ΔNLR		ΔPLR		ΔSII	
		*r*	*P*	*r*	*P*	*r*	*P*
6 months	ΔUTP	0.122	0.040	0.097	0.103	0.096	0.095
	ΔSCr	0.175	0.003	0.071	0.236	0.156	0.009
	ΔAlb	-0.033	0.580	-0.094	0.118	-0.211	0.001
	ΔrSLEDAI	-0.004	0.945	-0.015	0.798	-0.025	0.674
12 months	ΔUTP	0.143	0.019	0.098	0.107	0.165	0.007
	ΔSCr	0.158	0.010	0.090	0.141	0.174	0.004
	ΔAlb	-0.074	0.230	-0.115	0.064	-0.042	0.500
	ΔrSLEDAI12	0.038	0.537	0.023	0.708	-0.004	0.950

We further analyzed the correlation of baseline NLR, PLR, and SII with the renal pathological types. Among the 159 patients who underwent renal biopsy, those with proliferative or mixed lupus nephritis (classes III/IV ± V) had a median AI of 7 (5, 10) and a CI of 2 (1, 3). However, no significant differences were observed between NLR, PLR, or SII and histopathological subtypes, as summarized in **Table 4**. We then categorized the lupus nephritis pathological types into proliferative and non-proliferative lupus nephritis, but still found no significant difference in NLR, PLR, SII between proliferative and non-proliferative types of LN (p all>0.05) (**Table 5**).

**Table 4:** Correlation between baseline NLR, PLR, SII and LN types.

Systemic inflammatory markers	III type (n=17)	IV type (n=52)	V type (n=30)	III+V type (n=20)	IV+V type (n=34)	Other types (n=6)	p values
NLR	4.5 (3.4, 7.1)	4.2 (2.8, 8.8)	5.5 (2.8, 8.6)	3.9 (2.3, 6.7)	6.0 (4.3, 8.8)	3.4 (2.1, 5.5)	0.181
PLR	188.8 (151.5, 271.9)	214.9 (123.5, 340.3)	197.7 (129.0, 341.7)	206.3 (120.7, 269.7)	195.7 (146.8, 327.7)	191.8 (142.6, 316.7)	0.991
SII	843.9 (625.6, 1947.5)	654.5 (368.1, 1783.7)	1110.5 (523.0, 2054.7)	1107.5 (413.2, 1452.0)	1020.3 (611.5, 2435.5)	792.6 (421.5, 1141.3)	0.556

**Table 5:** Comparation of NLR, PLR, SII between proliferative and non-proliferative LN/.

Systemic inflammatory markers	Proliferative LN (n=124)	Non-proliferative LN (n=35)	p values
NLR	4.8 (3.2, 7.7)	3.7 (2.7, 7.9)	0.464
PLR	203.6 (137.2, 304.8)	197.5 (132.9, 340.0)	0.795
SII	899.7 (352.2, 1911.2)	878.8 (516.0, 2009.3)	0.848

In addition, to explore the relationship between systemic inflammatory markers and renal pathologic activities, we further analyzed baseline NLR, PLR, and SII in relation to the AI and CI of lupus nephritis pathology. The results showed that NLR and SII were positively correlated with the AI score of renal pathology (NLR: *r* = 0.244, p=0.013; SII: *r* = 0.199, p=0.043), indicating the value in reflecting the disease activity of renal pathology (**Table 6**).

**Table 6:** Correlation between baseline NLR, PLR, SII and renal pathologic activities.

Renal pathological index	NLR		PLR		SII	
	*r*	*P*	*r*	*P*	*r*	*P*
Activity Index (AI)	0.244	0.013	0.129	0.194	0.199	0.043
Chronicity Index (CI)	-0.043	0.665	-0.080	0.425	-0.040	0.689

### Correlation between baseline NLR, PLR, SII and LN remission at 6 and 12 months

We compared the baseline NLR, PLR, SII of patients who achieved renal remission and those who did not at 6 months and 12 months. However, the comparisons did not yield statistically significant differences in baseline NLR, PLR or SII among the various renal remission states at either 6 or 12 months (**Table 7**).

**Table 7:** Comparison of baseline NLR, PLR, and SII among different renal remission states.

Times	Groups	n	NLR	p values	PLR	p values	SII	p values
6 months	CRR group	140	4.4 (3.0, 7.5)	0.729	178.9 (128.6, 298.9)	0.402	703.1 (394.0, 1358.4)	0.153
	Non-CRR group	162	4.9 (3.0, 7.6)		196.1(128.4, 309.6)		847.7 (453.2, 1472.1)	
	TRR group	206	4.7 (3.0, 7.6)	0.443	187.0 (126.4, 296.3)	0.449	775.3 (425.1, 1442.2)	0.908
	Non-TRR group	96	4.3 (2.7, 7.2)		201.6(131.3, 318.8)		817.9 (431.3, 1443.0)	
12 months	CRR group	172	4.5 (3.0, 7.2)	0.686	181.8(121.1, 295.6)	0.106	748.9 (394.5, 1334.6)	0.165
	Non-CRR group	112	5.0 (2.8, 8.2)		201.6 (138.5, 324.3)		878.3 (490.0, 1538.1)	
	TRR group	212	4.6 (3.0, 7.5)	0.354	185.9 (121.7, 297.8)	0.141	789.8 (429.9, 1388.2)	0.942
	Non-TRR group	72	4.2 (2.4, 8.2)		213.5 (148.6, 318.8)		917.5 (416.5, 1487.1)	

### Risk factors for TRR attainment at 6 months

To further elucidate the risk factors for achieving TRR at 6 months, logistic regression analysis was conducted. In the univariate analysis, factors associated with prolonged SLE duration [HR 0.947 (0.915, 0.981), p=0.002] and elevated complement C4 levels [HR 0.013 (0.001, 0.382), p=0.012] were adverse factors for renal remission. Conversely, the use of mycophenolate [HR 3.007 (1.531, 5.906), p=0.001] and HCQ [HR 1.890 (1.016, 3.516), p=0.044]were identified as favorable prognostic factors for TRR at 6 months.

For the multiple logistic regression analysis, we included all factors with p-values less than 0.1. Application of mycophenolate emerged as a significant prognostic factor [HR 2.147 (1.052, 4.384), p=0.036], while prolonged SLE duration remained a negative predictive factor for TRR at 6 months [HR 0.945 (0.907, 0.984), p=0.006] (**Table 8**).

**Table 8:** Risk factors associated with TRR at 6 months by logistic analysis.

Items	Univariate analysis		Multivariate analysis	
	HR (95% CI)	p value	HR (95% CI)	P value
Male sex	0.849 (0.430, 1.677)	0.638		
Age of onset SLE (years)	1.003 (0.985, 1.022)	0.740		
Age at enrollment (years)	0.989 (0.91, 1.006)	0.209		
SLE duration (years)	0.947 (0.915, 0.981)	0.002	0.945 (0.907, 0.984)	0.006
Hypertension	1.246 (0.740, 2.097)	0.408		
LN as the initial manifestation	0.685 (0.408, 1.152)	0.154		
Anti-dsDNA positive	0.886 (0.466, 1.686)	0.713		
Complement C3 (g/L)	0.388 (0.130, 1.160)	0.090	0.516 (0.104, 2.553)	0.417
Complement C4 (g/L)	0.013(0.001, 0.382)	0.012	0.101 (0.001, 12.581)	0.351
Scr (umo/L)	0.998 (0.994, 1.001)	0.130		
Alb (g/L)	1.010 (0.973, 1.049)	0.595		
UTP (g)	1.012 (0.995, 1.073)	0.682		
Hematuria	0.900 (0.544, 1.488)	0.680		
Pyuria	0.722 (0.444, 1.174)	0.189		
Cylindruria	0.752 (0.437, 1.294)	0.304		
Usage of mycophenolate	3.007 (1.531, 5.906)	0.001	1.008 (0.514, 1.975)	0.800
Usage of cyclophosphamide	1.247 (0.645, 2.410)	0.512		
HCQ usage	1.890 (1.016, 3.516)	0.044	2.147 (1.052, 4.384)	0.036
RASi usage	0.803 (0.477,1.351)	0.408		
SLEDAI-2K	1.004 (0.965, 1.045)	0.835		
WBC at baseline	1.081 (0.999, 1.170)	0.054	1.006 (0.921, 1.099)	0.890
HGB at baseline	1.002 (0.992, 1.014)	0.655		
PLT at baseline	1.001 (0.998, 1.004)	0.627		
NLR at baseline	1.040 (0.988, 1.095)	0.129		
PLR at baseline	1.000 (0.998, 1.001)	0.591		
SII at baseline	1.000 (1.000, 1.000)	0.072	1.000 (1.000, 1.000)	0.800
NLR at 3 months	1.017 (0.932, 1.110)	0.669		
PLR at 3 months	1.000 (0.998, 1.003)	0.932		
SII at 3 months	1.000 (1.000, 1.000)	0.383		
ΔNLR during 3 months	1.036 (0.983, 1.091)	0.186		
ΔPLR during 3 months	1.000 (0.998, 1.001)	0.553		
ΔSII during 3 months	1.000 (1.000, 1.000)	0.255		

## Discussion

This study described the changes in complete blood count parameters and systemic inflammatory indices (NLR, PLR, SII) over a 12-month period during the induction of remission in patients with LN. We observed significant increases in lymphocyte, platelet, and hemoglobin levels during the first three months, after which these parameters stabilized. In contrast, white blood cell (WBC) and neutrophil counts exhibited a trend of initially rising before subsequently decreasing back to baseline levels. Concurrently, NLR showed a gradual decline throughout the follow-up period, reflecting the effects of the induction therapy. The PLR was highest at baseline and decreased in subsequent follow-ups. However, the trend in SII was not pronounced.

This study was the first to explore the relationships between NLR, PLR and SII with LN laboratory indicators, renal remission, and renal pathology in a longitudinal cohort during the induction phase of LN. We found that NLR and SII are positively correlated with urine protein, serum creatinine and the activity index (AI) of renal pathology. Furthermore, the changes in NLR and SII are positively correlated with the changes in UTP and SCr, suggesting that NLR and SII, as systemic inflammatory markers, can reflect the severity of the disease and acute inflammatory status in patients with lupus nephritis.

However, it should be noted that the strength of the observed correlations, while statistically significant, was modest. This may be partly attributable to the well-documented phenomenon of clinical-histological dissociation in lupus nephritis, wherein the severity of clinical manifestations does not always correspond directly to the degree of histopathological activity (13). The complex and heterogeneous nature of LN implies that biomarkers such as NLR, PLR, and SII likely capture broad aspects of systemic inflammation, which may not fully align with specific renal pathological changes.

Mechanistically, these correlations may reflect integrated inflammatory pathways in LN: Neutrophil hyperactivation promotes glomerular injury via NETosis, releasing autoantigens that trigger interferon responses and complement deposition (14, 15). Concurrent lymphopenia impairs immunoregulation (16), while platelets (key to SII) amplify damage through microthrombosis and TGF-β-driven fibrosis (17, 18). Collectively, NLR/SII capture a systemic pro-thrombotic/pro-inflammatory state, explaining their association with histological activity.

NLR, PLR, and SII are inflammatory markers derived from different blood cell counts that reflect the systemic level of inflammation and disease activity in various rheumatic and autoimmune diseases. These indices have garnered attention in recent years as potential biomarkers for evaluating disease progression and treatment response.

Previous studies have established correlations between NLR and disease activity, organ involvement, and infection within the context of rheumatic diseases. For example, elevated NLR values have been associated with increased disease activity in conditions such as psoriatic arthritis (19), rheumatoid arthritis (5), polymyalgia rheumatica (20) and Takayasu’s arteritis (7). Similarly, PLR has also been linked to disease severity and inflammatory activity across several autoimmune disorders, serving as a useful prognostic indicator (2).

In the field of lupus research, studies have shown that NLR and other systemic inflammatory indices are significantly higher in lupus patients compared to healthy controls (8, 21–23). Furthermore, NLR levels are elevated in active lupus patients compared to those in a non-active state (24). There is a positive correlation between NLR and markers such as erythrocyte sedimentation rate, C-reactive protein, anti-dsDNA antibody titers, complement levels, and SLEDAI, indicating that NLR may provide valuable insights into lupus disease activity (8, 10, 23–26).

Regarding organ involvement, studies found that NLR was higher in patients with serositis. In contrast, PLR was elevated in lupus patients who presented with rashes, arthritis, or positive anti-Sm antibodies, while it was lower in patients with hematological involvement (27). Notably, patients with lupus nephritis exhibited significantly higher NLR levels compared to those without renal involvement (21, 25, 26). Additionally, there were statistically significant differences in NLR levels among different types of lupus nephritis (28). Our study found no significant statistical differences in NLR, PLR, and SII among different pathological types of LN.

Some studies reported that NLR and PLR levels were higher in lupus patients with infections compared to those without infections, suggesting that these indices could be valuable for distinguishing infectious complications in lupus patients (29). Other research explored the relationship between systemic inflammatory indices and patient-reported outcomes in SLE, revealing that patients in the high NLR group had a higher incidence and severity of depression (30). Moreover, it was found that SLE patients with high NLR tended to have a greater usage of corticosteroids and immunosuppressants (22).

Our study has several limitations. Firstly, glucocorticoids can significantly influence hematological parameters, particularly levels of white blood cells and neutrophils. Although we made efforts to obtain complete blood count data prior to the initiation of induction therapy, some relapse patients had already been receiving glucocorticoids. Furthermore, during the entire follow-up period, the majority of patients continued glucocorticoid therapy, albeit at gradually decreasing doses as their condition improved. Secondly, immunosuppressants may also impact complete blood count results. While we excluded cases with evident bone marrow suppression, it remains inevitable that immunosuppressants such as cyclophosphamide could lead to mild bone marrow suppression. Lastly, it is important to acknowledge that renal biopsy was not performed in all patients, and consequently, activity and chronicity indices were not available for the entire cohort. This may introduce potential selection bias and limit the generalizability of the histopathological correlations presented in this study. Nonetheless, this reflects real-world clinical practice in which renal biopsy is not always feasible due to contraindications, patient preference, or limited medical resources.

## Conclusion

Our study is the first to describe the trends in complete blood count parameters and systemic inflammatory indices in a longitudinal cohort of LN patients undergoing induction therapy. We found that NLR and SII are positively correlated with urine protein levels, serum creatinine, and the activity index (AI) of renal pathology, underscoring their significance in reflecting the severity and activity of lupus nephritis. These systemic inflammatory indices provided us a quick, simple and effective supervision way in monitoring and managing LN patients.

## Data Availability

The raw data supporting the conclusions of this article will be made available by the authors, without undue reservation.
