# Supplementary material for: NLR (neutrophil to lymphocyte ratio), PLR (platelet to lymphocyte ratio), and SII (systemic immune-inflammation index) reflect disease activity and renal remission in patients with lupus nephritis
Source: Front Immunol. 2025 Sep 30;16:1646276. doi: 10.3389/fimmu.2025.1646276 (PMC12518351; doi:10.3389/fimmu.2025.1646276)
Supplement: Supplementary file 1 [file Table1.docx]

| Sup table1. Baseline clinical characteristics of enrolled patients | |
| --- | --- |
| **Clinical data** | **Total patients (n=310)** |
| Age of SLE onset (years) | 30.9±13.3 |
| Age at enrollment (years) | 35.9±14.2 |
| SLE duration (years) | 2.1 (0.3, 7.2) |
| Female, n (%) | 264 (85.2%) |
| Initial treatment, n (%) | 163 (52.6%) |
| Clinical Manifestations |  |
| Fever | 81 (26.1%) |
| Rash | 122 (39.4%) |
| Alopecia | 59 (19.0%) |
| Oral ulcers | 31 (10.0%) |
| Arthritis | 86 (27.7%) |
| Myositis | 24 (7.7%) |
| Serositis | 65 (21.0%) |
| Lupus nephritis | 310 (100%) |
| Neuropsychiatric SLE | 20 (6.5%) |
| Hematological involvement |  |
| Hemolytic anemia | 44 (14.2%) |
| Leukopenia | 85 (27.4%) |
| Thrombocytopenia | 46 (14.8%) |
| Laboratory data |  |
| UTP (g) | 3.1 (1.5, 5.4) |
| Haematuria, n (%) | 196 (63.2%) |
| Pyuria, n (%) | 155 (50.0%) |
| Urinary casts, n (%) | 94 (30.3%) |
| SCr (μmol/L) | 74.0 (61.2, 100.0) |
| Alb (g/L) | 28.0±6.5 |
| ANA positive, n (%) | 300 (100%) |
| Anti-dsDNA positive, n (%) | 258 (83.2%) |
| Anti-nRNP positive, n (%) | 131 (42.3%) |
| Anti-Sm positive, n (%) | 105 (33.9%) |
| Anti SSA positive, n (%) | 180 (58.1%) |
| Anti SSB positive, n (%) | 52 (16.8%) |
| Anti rRNP positive, n (%) | 89 (28.7%) |
| ANCA positive, n (%) | 29 (9.4%) |
| Lupus anticoagulants positive, n (%) | 48 (15.5%) |
| Anticardiolipin antibodies positive, n (%) | 32 (10.3%) |
| Antibodies against β 2 Glycoprotein I positive, n (%) | 48 (15.5%) |
| Complement C3 (g/L) | 0.46 (0.32, 0.58) |
| Complement C4 (g/L) | 0.09 (0.05, 0.13) |
| SLEDAI-2K | 17.1±6.1 |
| Treatment strategies |  |
| Prednisolone pulse, n (%) | 43 (13.9%) |
| Initial Prednisolone, (mg/d) | 58.5±17.8 |
| Categories of immunosuppresants: MMF/CYC/others, n | 140/115/55 |
| HCQ usage, n (%) | 259 (83.5%) |
| Note: WBC (×109/L): white blood cell; HGB (g/L): hemoglobin; PLT (×109/L): platelets; UTP (g): 24 hours urinary protein; SCr: serum creatinine; Alb: serum albumin; SLEDAI: SLE disease activity index; HCQ: hydroxychloroquine; MMF: mycophenolate; CYC: cyclophosphamide; | |
